# Supplementary material for: Vitamin D Modified DSS-Induced Colitis in Mice via STING Signaling Pathway
Source: Biology (Basel). 2025 Jun 18;14(6):715. doi: 10.3390/biology14060715 (PMC12190092; doi:10.3390/biology14060715)
Supplement: Supplementary file 1 [file biology-14-00715-s001.zip › Supplementary Materials S1: 16s-report of gut microbiota/Groups/diff_analysis/RF/KEGG_Groups.RFimportance.pdf]

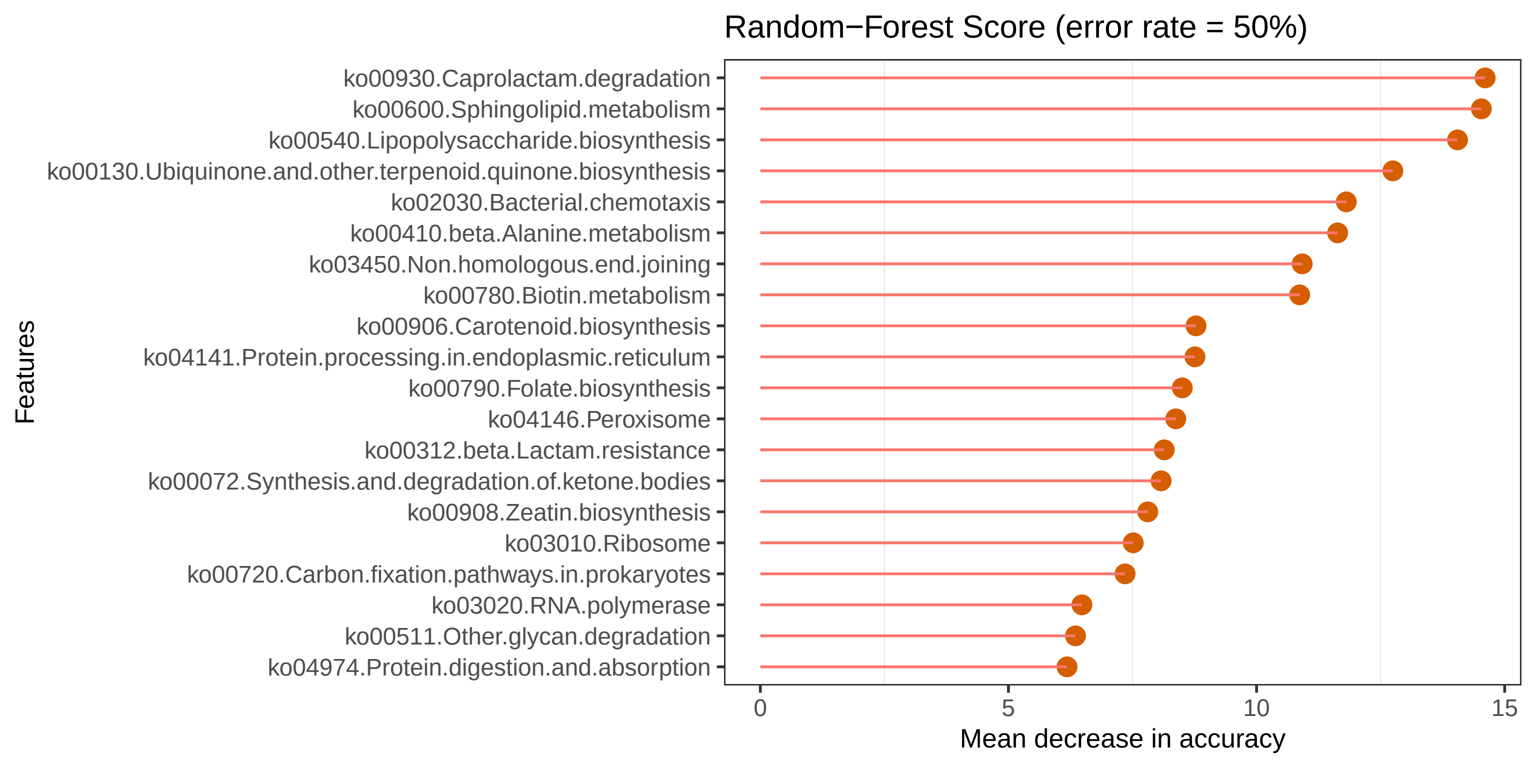

Random−Forest Score (error rate = 50%)
Features
ko00930.Caprolactam.degradation
ko00600.Sphingolipid.metabolism
ko00540.Lipopolysaccharide.biosynthesis
ko00130.Ubiquinone.and.other.terpenoid.quinone.biosynthesis
ko02030.Bacterial.chemotaxis
ko00410.beta.Alanine.metabolism
ko03450.Non.homologous.end.joining
ko00780.Biotin.metabolism
ko00906.Carotenoid.biosynthesis
ko04141.Protein.processing.in.endoplasmic.reticulum
ko00790.Folate.biosynthesis
ko04146.Peroxisome
ko00312.beta.Lactam.resistance
ko00072.Synthesis.and.degradation.of.ketone.bodies
ko00908.Zeatin.biosynthesis
ko03010.Ribosome
ko00720.Carbon.fixation.pathways.in.prokaryotes
ko03020.RNA.polymerase
ko00511.Other.glycan.degradation
ko04974.Protein.digestion.and.absorption
0
5
10
15
Mean decrease in accuracy
